# The Application of Biosorption for Production of Micronutrient Fertilizers Based on Waste Biomass

**DOI:** 10.1007/s12010-014-1074-0

**Published:** 2014-08-10

**Authors:** Łukasz Tuhy, Mateusz Samoraj, Izabela Michalak, Katarzyna Chojnacka

**Affiliations:** 1 Institute of Inorganic Technology and Mineral Fertilizers, Wrocław University of Technology, Smoluchowskiego 25, 50-372 Wrocław, Poland; 2 Gdańska 7/9, 50-344 Wrocław, Poland

**Keywords:** Waste biomass, Biosorption, Zinc, Micronutrient fertilizers, Bioavailability

## Abstract

In the present paper, new environmental-friendly fertilizer components were produced in biosorption process by the enrichment of the biomass with zinc, essential in plant cultivation. The obtained new preparations can be used as controlled release micronutrient fertilizers because microelements are bound to the functional groups present in the cell wall structures of the biomass. It is assumed that new fertilizing materials will be characterized by higher bioavailability, gradual release of micronutrients required by plants, and lower leaching to groundwater. The biological origin of the material used in plant fertilization results in the elimination of toxic effect towards plants and groundwater mainly caused by low biodegradability of fertilizers. Utilitarian properties of new formulations enable to reduce negative implications of fertilizers for environmental quality and influence ecological health. In this work, the utilitarian properties of materials such as peat, bark, seaweeds, seaweed post-extraction residues, and spent mushroom substrate enriched via biosorption with Zn(II) ions were examined in germination tests on *Lepidium sativum*. Obtained results were compared with conventional fertilizers—inorganic salt and chelate. It was shown that zinc fertilization led to biofortification of plant in these micronutrients. Moreover, the mass of plants fertilized with zinc was higher than in the control group.

## Introduction

Intensive exploitation of soils causes many problems, i.e., microelement impoverishment. Trace metals play an important role in plant nutrition. They are required in very small quantities by plants. However, the proper growth of plants without trace elements would be impossible. Microelements act as cofactors and participate in various metabolic pathways [1]. Trace elements are also necessary for the proper functioning of the processes of photosynthesis and respiration. Currently, new varieties of plants, i.e., maize and wheat are particularly sensitive to deficiency of trace elements in the soil [2]. This results in impaired growth and development and leads to decrease in crop yield. Total level of micronutrients does not reflect their bioavailability to plants [2]. Bioavailability is defined as the amount of nutrients that is available to plant in a useful form and it depends on soil type, content of organic matter, pH, adsorptive surface, and other chemical, physical, and biological conditions [2, 3]. The bioavailability of different forms of trace elements in soil can be tested by in vivo tests on plants. Bioavailability can be measured by in vitro tests and in vivo by direct uptake experiments by plants. Laboratory in vitro tests of bioavailability are assessed by chemical extraction, membrane techniques (passive samplers), or isotope dilution techniques. In vivo tests are used to determine quantity of metal ions taken up by organisms (i.e., in germination tests) [4]. Metal ions extracted chemically in vitro can be correlated with in vivo test results (plant uptake). Correlation between ion concentration in plant and soil is expressed as transfer factor (TF), which is the quotient of these values [2, 5] and can be expressed as percentage.

Micronutrient deficiency can be overcome by micronutrient fertilization. There are many different fertilizers such as chelates and inorganic technical salts of micronutrients available on the market. Chelates are characterized by high bioavailability, but they are quite expensive [6]. Technical salts are low-cost fertilizers but micronutrients are easily dissolved in percolating water and are not released in controlled way.

Among micronutrients, zinc plays a very important role in growing of plants. It is a part of the RNA polymerase and constitutes the activator of enzymes correlated with metabolism of proteins and carbohydrates. In addition, zinc ions stabilize the protein structure and are involved in gene expression. Deficiency of zinc in plants can lead to discoloration of the leaves, as well as size reduction. Plants absorb zinc from soil in the following forms: ions of Zn^2+^, organic-zinc complexes, and exchangeable forms from soil colloidal fraction. Less available are forms from insoluble complexes [7]. The proper fertilization of plants can prevent micronutrient deficiency and thus increases their nutritional value. Micronutrient fertilizers with zinc should be characterized by high bioavailability of zinc, which means high uptake by plants.

Zinc(II) ions can be bound to low-cost biological materials by biosorption. As it was previously shown, biosorption process can be used not only for removal of toxic pollutants from wastewaters but also to enrich the biomass with trace elements which are essential in plant nutrition [8]. During biosorption, metabolically inactive organic matter is enriched with micronutrients in a process based mainly on adsorption and ion exchange. Metal ions can bind with high affinity to different functional groups such as carboxyl, amine, hydroxyl, etc. present on cell walls. Low costs of materials, easy handling equipment, and high efficiency are the major advantages of biosorption [9]. The main advantages of biological components are biodegradability, lack of toxic effect, and low price. Among organic raw materials, many of them can be used as biosorbents. Moreover, enriched biomass can also release metal ions in extraction process in a controlled way according to equilibrium dependence.

The aim of this work was to evaluate the possibility of the application of different biomasses (peat, bark, seaweeds, seaweeds post-extraction residues, and spent mushroom substrate) enriched with zinc(II) ions as fertilizers with micronutrients. For this purpose, the bioavailability of zinc(II) ions from biological fertilizer components and the influence of different enriched biomasses on element content of plant and plant yield compared with conventional fertilizers with micronutrients were examined.

## Methods

### Sample Preparation

For the biosorption experiments, five biomaterials were used: commercially available peat—“Horticultural peat—Athena,” garden bark (“Pine bark—Athena”), spent mushroom substrate obtained from mushroom farm “HAJDUK,” seaweeds collected from Baltic coasts during summertime, and post-extraction residues after supercritical CO_2_ extraction conducted on those seaweeds. The biosorption of zinc(II) ions by biological materials was carried out in batch mode in stirred tank reactors (60 L). The concentration of Zn(II) ions (as ZnSO_4_ · 7H_2_O (Chempur, Poland)) in the solution was 300 mg/L, pH 5 measured in 25 °C with the use of pH meter Mettler Toledo SevenMulti, Switzerland. The concentration of the biomass was 1.0 g of dry mass (DM)/L according to our previous studies [10]. The mixture was stirred by aerating with the air pump for an hour. The solution was then filtered, and the biomass was air-dried. The content of elements in the enriched biomass was examined by ICP-OES.

### Germination Tests

The aim of these experiments was to evaluate the effect of different types of biomass enriched with Zn(II) ions in biosorption process on the germination of seeds, when compared with the control groups—deionized water, chelate, and inorganic salt. Dose of Zn(II) used in the experiment was 4.0 mg/petri dish for each fertilizer. Seven types of fertilizers were tested: biomaterials enriched with Zn(II) ions—seaweeds (seaweeds-Zn), post-extraction residues (residues-Zn), spent mushroom substrate (SMS-Zn), pine bark (bark-Zn), peat (peat-Zn), Zn–EDTA chelate (Symfonia® Zn, Anwil, Poland), and ZnSO_4_ · 7H_2_O (Chempur, Poland). To compare the fertilizing properties, germination tests were performed on garden cress *Lepidium sativum* according to the international norm (the International Seed Testing Association). Plastic dishes with the cotton (approximately 5.0 g) soaked with deionized water were prepared. On each dish, 50 seeds were placed in rows at equal distances from each other. In the next step, seeds were subjected to the stratification in 1 °C for 3 days. After stratification, appropriate amounts of particular fertilizer were spread evenly on Petri dishes. Each probe was taken in triplicates. Germination tests were performed in seed germinator (Jacobsen J120/OS) in 25 °C for 10 days after stratification. Plants were watered with 5 mL of deionized water every day during experiment. After experiment, the plant biomass from each plate was dried to the constant weight and mineralized and multielemental analysis by ICP-OES was carried out (three measurements of each material).

### Analytical Methods

Seaweeds-Zn, residues-Zn, peat-Zn, SMS-Zn, bark-Zn (0.5 g), and plants after germination (whole yield) were purified from organic matter with concentrated nitric acid—69 % m/m (5 mL), spectrally pure (Suprapur, Merck, USA) in teflon bombs in microwave oven Milestone Start D (USA). The selected parameters of the process assured the complete digestion of samples. After mineralization, samples were diluted ten times with redemineralized water (Millipore Simplicity) and underwent multielemental analysis. The concentration of elements in digested biomass was determined by ICP-OES Varian-Vista MPX (Australia), equipped with ultrasonic nebulizer CETAC U5000AT+. The analyses were carried out in Laboratory Accredited by Polish Centre of Accreditation (PCA) according to PN-EN ISO/IEC 17025:2005. Quality assurance of the test results was achieved by using Combined Quality Control Standard from ULTRA Scientific, USA. The samples were analyzed in three repeats (the reported results of analyses were arithmetic mean, the relative standard deviation was <5 %).

### RGB Analysis

Microscopic images of plants (five images of different areas of leaf performed for each experimental group) were taken using a microscope (Carl Zeiss Axio Scope. A1), and the analysis of intensity of red, green, and blue colors of leaves was examined using RGB model in computational application AxioVisionRelease 4.8. RGB model enables to evaluate the intensity of red, green, and blue color and therefore determine chlorophyll content in leaves.

### Statistical Analysis

Obtained results were statistically elaborated using Statistica software ver. 9.0. For all obtained results, the distribution was tested for normality test (Shapiro–Wilk). For normal distribution, homogeneity of variance was checked using Brown–Forsyth test. In the groups which fulfilled the condition of homogeneity of variance, *F* test of analysis of variance was conducted. For other than normal distribution, Kruskal–Wallis test was carried out to find statistically significant differences between tested groups. It was assumed that the results were statistically significant at *p* < 0.05. For groups characterized by normal distribution, the mean value was calculated, while for other than normal distribution, the median was reported.

## Results and Discussion

Seaweeds, bark, peat, and spent mushroom substrate are examples of biomass that can be used as a biological fertilizer component. About 500 tons of seaweeds are collected every year only in Sopot (Poland). Utilization of similar amounts of biomass from other parts of the coast in Poland constitutes a serious problem. Globally, over 2 million tons of seaweeds is harvested from seacoast every year. One of utilization possibilities is usage as biological component in fertilization of plants [11].

Another promising low-cost biosorbent is pine bark [12]. Bark is a by-product of the timber industry, demand for which is low. This material is often used as fertilizer component which improves soil structure and may act as biosorbent [12, 13]. The bark can be used to purify water from metal ions [13]. Studies have shown that it can be used in metal ion removal from rain water and other solutions [14].

Also, peat can be used as a biosorbent with many advantages. It is cheap, widely available, and has high buffering capacity [15]. Surface of peat is very complex with high porosity. Moreover, the main constituents of the surface of peat are lignins and humic acids which contain various functional groups as carboxylic and phenolic acids. The metal ion binding is mainly dependent on pH. In low pH, uptake of anions is favored, while in higher pH, more effective is biosorption of cations. Studies have shown that optimum pH for biosorption of most divalent ions onto peat is in the range 3.5 to 6.5 [16] .

Spent mushroom substrate (SMS) improves soil structure and quality—it is also a good biosorbent. The production of each kilogram of mushrooms generates about 5 kg of this waste material [17, 18]. SMS improves soil structure and provides plant-available nutrients. Two main types of SMS are currently used in industry. SMS-AB and SMS-P are subsoils after cultivation of *Agaricus bisporus* and *Pleurotus*, respectively. SMS-AB contains composted straw and muck, soil, CaSO_4_, and inorganic matter (nutrients, pesticides). SMS-P is composed of fermented straw and inorganic matter (nutrients, pesticides). SMS-AB has similar or higher content of organic substances, Ca, nutrient content as organic fertilizers—therefore, soil application can be used as a recycling strategy [18]. Fresh SMS contains high salt concentration and may be used as a growing medium for salt sensitive plants. Some studies suggest that SMS can be used as an independent, soil-less medium [17].

The aim of the present study was to evaluate the effect of enriched with Zn(II) ion biomass (mentioned above) on the germination of *L. sativum* and the mineral content of the germinated plants.

### Elemental Composition of Germinated Plants

The mineral composition of each plant fertilized with different components was determined in the present study. Statistically significant differences in element content between plants fertilized with different materials were also investigated. Each fertilizer component was tested in triplicates. The plant yield was collected separately from dishes, and ICP-OES analysis were carried out in triplicates (*N* = 9). Tables 1 and 2 present results of the levels of elements in the case of which statistically significant differences between the tested groups were observed. As it was shown, the application of biological fertilizer components increased the content of important nutrients in plants, i.e., Fe and Cu, in comparison with the control group about 31–123 % and 2–98 %, respectively. Statistically significant differences in the level of B which is structural element of plant cell wall [19] and Si (inducing broad spectrum of disease resistance in plants) [20], the contents were also observed in plants fertilized with different materials with zinc(II). Increased content of Al in plants fertilized with all the components was also observed. It caused the reduction of calcium uptake and slight reduction of Mg content that can result in increased toxicity to plants [21], but it did not lead to the reduction of plant yield. Furthermore, aluminum stress which is revealed by the decrease of potassium content [22] was not observed in the present study. The application of SMS-Zn, seaweeds-Zn, and residues-Zn resulted in increased content of Cu in plants (twice as high as in control group) while commercial fertilizers caused the reduction of Cu content in comparison with nonfertilized group. The same observation was made for Fe content, the shortage of which can cause chlorosis and inhibit plant growth [23] and can constitute another explanation to the differences in plant mass between groups fertilized with SMS-Zn, seaweed-Zn, residues-Zn, peat-Zn, and other groups.

**Table 1:** The difference in the content of elements expressed as the mean in plants fertilized with different components (normal distribution, *N* = 9)

	The content of elements in plant (mg/kg DM) after germination tests															
	Control		Zinc sulfate		Zn-EDTA		Bark-Zn		SMS-Zn		Seaweeds-Zn		Residues-Zn		Peat-Zn	
	$\overline{x}$	SD	$\overline{x}$	SD	$\overline{x}$	SD	$\overline{x}$	SD	$\overline{x}$	SD	$\overline{x}$	SD	$\overline{x}$	SD	$\overline{x}$	SD
Al	9.810^A^	2.520	12.47^B^	12.39	11.58^C^	5.32	22.08^D,a^	5.74	65.61^A,B,C,D,E,F^	28.20	31.74^E^	3.51	31.69^F^	7.11	54.47^a^	6.59
B	141.6^A,B,C,D^	15.7	160.5^E^	20.3	177.7	34.8	215.9^A,E^	17.0	200.9^B^	20.4	199.8^C^	17.7	208.8^D^	18.5	187.1	7.1
Ca	2,027^A^	39	1,884	78	1,560^C,a^	278	2,527^D,a^	72	5,823^A,B,C,D,E,F,G^	925	1861^E^	214	1,909^F^	33	1718^G^	147
Cu	7.540^A,E,I^	0.972	6.021^B,F,J^	0.450	6.140^C,G,K^	0.860	6.220^D,H,L^	0.640	14.92^A,B,C,D,M^	0.23	12.38^E,F,G,H,N^	4.07	12.49^I,J,K,L,O^	1.53	7.710^M,N,O^	0.434
Fe	111.2^A,E^	19.3	96.36^B,F^	8.90	111.4^D,H^	11.4	111.4^D,H^	11.4	247.9^A,B,C,D,I,J,a^	35.0	181.5^E,F,G,H,a^	45.3	148.2^I^	17.7	145.3^J^	8.9
K	14,832	855	12,751^A^	654	13,688	2,000	17,298^A,a^	274	16,022	744	13,864^a^	1,134	14,765	351	14,329	2,496
Si	48.20^A^	3.61	31.67^B^	6.25	30.89^C^	6.28	44.28^D^	0.65	144.3^A,B,C,D,E,F,G^	38.8	69.21^E^	1.89	47.68^F^	8.41	47.31^G^	2.16

*A*, *B*… *p* < 0.05; *a p* < 0.1

**Table 2:** The difference in the content of elements expressed as the median in plants fertilized with different components (other than normal distribution, *N* = 9)

	The content of elements in plant (mg/kg DM) after germination tests															
	Control		Zinc sulfate		Zn-EDTA		Bark-Zn		SMS-Zn		Seaweeds-Zn		Residues-Zn		Peat-Zn	
	Median	SD	Median	SD	Median	SD	Median	SD	Median	SD	Median	SD	Median	SD	Median	SD
Li	0.2202^A,B^	0.0232	0.0991^A^	0.0238	0.0733^B^	0.0256	0.1884	0.0149	0.1375	0.0174	0.1750	0.0377	0.1708	0.0080	0.1350	0.0259
Mn	29.14^A^	0.04	27.38	1.43	26.67^A^	4.22	48.30	9.69	44.54	3.96	29.59	2.23	31.01	0.32	30.20	2.83
Na	5,874^A,a^	335	2,563^A^	303	2918^a^	687	4,170	415	3,316	149	3,850	1,557	3,748	246	3,657	407
Zn	131^A,B^	9	1,913	217	1,375	247	582.9	241.0	2,302^A^	184	2,157^B^	105	2,021	242	1,858	169

*A, B… p* < 0.05; *a p* < 0.1

No statistically significant differences were observed for Mn and Na content in plants between new preparations and commercial products.

Enrichment of the biomass with trace elements can be also applied in biofortification of food and can constitute an instrument in human nutrition. The main function of micronutrients is to serve as prosthetic groups, activators of enzymatic reactions. and in metalloproteins in human organism. and its deficiency can result in serious health problems often in the form of hidden hunger [1, 24]. In the developing countries, this problem occurs among women and children. They suffer mainly for deficiencies of zinc and iron. More than 30 % of the world’s population suffers for zinc deficiency [24, 25]. The third deficient micronutrient in more than 0.5 billion people is selenium mainly in developed countries [26].

### Calculation of Transfer Factor

For quantitative description of zinc bioavailability from fertilizers to plant, transfer factor was calculated. Each fertilizer component was tested in triplicates. Biomass from each Petri dish was separately collected and analyzed by ICP-OES method in three replicates.

In the present study, it was observed that zinc fertilization with the use of different fertilizer components led to biofortification of plant in this micronutrient. The effect of zinc fertilization on zinc content in plants is presented in Tables 2 and 3. The content of zinc in plants increased from 130.7 mg/kg DM for plants not treated with any components with Zn(II) to 2,302 mg/kg DM for plants fertilized with SMS-Zn (17.6 times higher).

**Table 3:** The difference in transfer factor (TF) in plants fertilized with different components (normal distribution, *N* = 9)

	Bioavailability of Zn(II) ions (%)															
	Control		Commercial preparations				New biosorption preparations									
			Zinc sulfate		Zn-EDTA		Bark-Zn		SMS-Zn		Seaweeds-Zn		Residues-Zn		Peat-Zn	
	Median	SD	Median	SD	Median	SD	Median	SD	Median	SD	Median	SD	Median	SD	Median	SD
TF	–	–	4.067	0.578	2.508^a^	0.438	1.093^A,B^	0.404	5.167^A,a^	0.439	4.605^B^	0.169	4.257	0.415	3.742	0.326

*A*, *B* p < 0.05; *a p* < 0.1

Efficient biofortification of *L. sativum* in zinc was also observed for seaweeds-Zn (2,157 mg/kg DM) and residues-Zn (2,021 mg/kg DM). The efficiency of zinc fertilization in other cases was lower, and the content of zinc in plants fertilized with the use of zinc sulfate, peat-Zn, Zn-EDTA, and bark-Zn was 1,913, 1,858, 1,375, and 582.9 mg/kg DM, respectively. Table 3 presents the differences in transfer factor (TF) between different biological fertilizer components and commercial fertilizers. The conducted experiments showed that zinc fertilization with the use of SMS-Zn is the most efficient while bark-Zn is characterized by the lowest bioavailability of Zn(II) ions. Fertilizer components—SMS-Zn and seaweeds-Zn—were shown to be much better source of Zn to plants than Zn-EDTA. Furthermore, three of the biological fertilizer components with zinc (seaweeds-Zn, residues-Zn, and SMS-Zn) showed higher bioavailability of zinc ions than zinc sulfate—other commercially used components of fertilizers. Each group differed also in the plant yield. The mass of plants fertilized with zinc was higher than in the control group (0.0717 g) in all cases beside Zn-EDTA (0.0706 g). The best results were obtained for SMS-Zn (0.0853 g), residues-Zn (0.0847 g), and seaweeds-Zn (0.0844 g). The masses of plants fertilized with zinc sulfate, peat-Zn, and bark-Zn were 0.0837, 0.0759, and 0.0729 g, respectively.

### Correlations

Eight experimental groups (all the preparations and the control group) were tested in triplicates. The biomass from each plate was collected separately, and multielemental composition was analyzed also in triplicates (*N* = 72). Basing on this analysis, correlation matrix was plotted and presented on Table 4. It was shown that the mass of plant was strongly correlated with zinc content in plant (*r* = 0.76).

**Table 4:** Correlation matrix for element content in germinated plants, the number of germinated seeds (GS), and mass of plant (MP) (*N* = 72, *p* < 0.05)

	Al	B	Ba	Bi	Ca	Cd	Co	Cr	Cu	Fe	K	Li	Mg	Mn	Mo	Na	Ni	Pb	Pd	Pt	S	Sb	Se	Si	Sn	Sr	Zn	Zr	GS	MP
Al	1.00																													
B	0.35	1.00																												
Ba	0.31	−0.05	1.00																											
Bi	−0.33	*−0.45*	−0.25	1.00																										
Ca	*0.64*	0.26	−0.12	−0.12	1.00																									
Cd	0.34	0.25	0.21	−0.31	0.26	1.00																								
Co	0.17	−0.06	−0.02	−0.19	0.25	−0.2	1.00																							
Cr	*0.44*	0.33	−0.13	−0.3	*0.46*	0.16	0.31	1.00																						
Cu	*0.57*	0.39	−0.08	−0.33	*0.58*	0.21	0.34	*0.96*	1.00																					
Fe	*0.79*	0.37	0.07	−0.38	*0.75*	0.28	0.31	*0.82*	*0.89*	1.00																				
K	0.27	*0.49*	−0.14	−0.17	*0.46*	0.02	0.31	0.11	0.21	0.31	1.00																			
Li	0.03	0.15	−0.06	0.04	0.06	*−0.46*	0.34	0.18	0.25	0.2	*0.53*	1.00																		
Mg	0.13	0.24	−0.06	−0.13	0.35	−0.38	0.31	0.07	0.17	0.27	*0.60*	*0.46*	1.00																	
Mn	0.34	*0.49*	−0.15	0.04	*0.60*	−0.12	0.18	0.14	0.23	0.37	*0.76*	0.38	*0.51*	1.00																
Mo	0.33	−0.15	0.12	−0.24	0.27	0.02	0.07	0.39	0.38	*0.52*	−0.03	0.14	0.18	0.01	1.00															
Na	−0.17	0.04	−0.15	−0.01	−0.11	−0.37	0.20	0.02	0.02	−0.02	0.22	*0.76*	0.19	0	0.01	1.00														
Ni	*0.52*	0.37	0.08	−0.06	*0.44*	0.24	0.33	*0.65*	*0.65*	*0.67*	0.13	0.03	0.15	0.25	0.29	−0.11	1.00													
Pb	0.39	0.27	0.24	−0.29	0.19	0.15	0.08	*0.68*	*0.68*	*0.60*	0.07	0.27	0.14	0.05	*0.55*	0.05	0.33	1.00												
Pd	*0.55*	0.08	−0.1	−0.25	*0.45*	0.08	*0.50*	*0.71*	*0.70*	*0.71*	0.16	0.07	0.24	0.12	0.33	−0.15	0.39	*0.52*	1.00											
Pt	−0.03	−0.4	0.06	0.20	−0.17	−0.25	−0.03	−0.17	−0.16	−0.12	−0.27	0.09	−0.01	−0.08	0.39	−0.01	0.08	−0.11	−0.18	1.00										
S	0.22	*0.54*	−0.03	−0.36	0.31	0.36	−0.17	−0.04	0.08	0.21	*0.46*	−0.07	*0.53*	0.30	−0.04	−0.12	0.15	−0.09	0.05	−0.17	1.00									
Sb	0.40	−0.03	−0.12	−0.05	0.27	−0.1	0.04	0.26	0.27	0.36	−0.13	−0.07	0.08	−0.05	0.26	−0.05	0.27	0.06	0.38	0.09	−0.05	1.00								
*Se*	−0.36	−0.19	−0.09	0.02	−0.1	*−0.43*	0.05	−0.21	−0.25	−0.25	0.01	0.08	0.29	−0.01	0.14	0.07	−0.16	−0.07	−0.27	0.23	−0.08	−0.27	1.00							
*Si*	*0.76*	0.27	−0.04	−0.23	*0.93*	0.30	0.30	*0.61*	*0.71*	*0.89*	0.34	0.14	0.26	*0.45*	0.34	0.02	*0.52*	0.36	*0.57*	−0.16	0.20	0.32	−0.19	1.00						
*Sn*	*0.59*	0.21	−0.07	−0.25	*0.87*	0.25	0.12	0.30	0.39	*0.60*	*0.45*	0.01	0.29	*0.53*	0.17	−0.13	0.13	0.12	0.40	−0.15	0.32	0.21	0.04	*0.82*	1.00					
*Sr*	*0.66*	0.15	*0.87*	−0.34	0.36	0.35	0.14	0.19	0.28	*0.49*	0.09	−0.03	0.10	0.14	0.22	−0.19	0.34	0.39	0.22	−0.09	0.11	0.08	−0.19	*0.45*	0.34	1.00				
*Zn*	*0.61*	0.35	0.18	−0.23	0.3	0.36	0	*0.59*	*0.56*	*0.61*	−0.21	*−0.41*	−0.04	−0.07	0.18	*−0.44*	*0.63*	*0.45*	*0.58*	−0.19	0.18	0.38	−0.3	*0.45*	0.21	*0.41*	1.00			
*Zr*	0.33	0.31	0.13	−0.28	*0.53*	0.34	0.13	0.37	*0.43*	*0.55*	*0.43*	−0.04	*0.42*	0.35	0.26	−0.19	*0.52*	0.32	0.31	−0.31	*0.47*	0.11	0.07	*0.46*	0.37	0.40	0.29	1.00		
*GS*	*0.57*	*0.67*	−0.04	−0.3	*0.42*	0.06	0.26	*0.67*	*0.71*	*0.66*	*0.52*	*0.49*	0.16	*0.55*	0.17	0.27	*0.53*	*0.55*	0.37	−0.16	0.04	0.11	−0.23	*0.53*	0.33	0.25	0.32	0.25	1.00	
*MP*	*0.49*	0.29	−0.08	−0.18	0.36	−0.04	0.05	*0.71*	*0.66*	*0.61*	−0.22	−0.14	0.02	0.02	0.27	−0.12	*0.53*	0.40	*0.55*	−0.11	−0.02	*0.59*	−0.28	*0.48*	0.24	0.18	*0.76*	0.11	*0.45*	1.00

Statistically important results are in italics

Moreover, the plant yield was the highest for components characterized by the highest TF for Zn(II) ions. Those results can confirm the hypothesis that zinc participates in plant growth hormone synthesis that was described in the literature [27].

Additionally, interdependencies between elements, the number of germinated seeds, the mass of germinated plants, and some statistically significant correlations are presented in Table 4. The main significant correlations of synergistic type found in this study are Cu/Cr > Ca/Si (*r* > 0.90); Fe/Cu, Fe/Si > Sn/Ca > Sr/Ba > Ti/Fe > Si/Sn > Fe/Cr (*r* > 0.80), and Fe/Al > Mn/K, Na/Li > Fe/Ca > Cr/Pd > Cu/Pd (*r* > 0.70). There are known some statistically important synergistic correlations in the literature [28], between Sr/Ba (r = 0.87) which was also found in our study. The alkali metals (i.e., K) and the alkaline earth metals (Ca, Mg) and Sr demonstrate high correlations between each other, as well as with the macronutrients N and P [29]. In our study, we proved the correlation between K/Ca (*r* = 0.46) and K/Mg (*r* = 0.60). The role of K, Mg, and Ca in plant metabolism and interdependencies between those three elements are also described in the literature [28]. Trivalent ions such as Al, Fe, and Sc have very similar radius of the hydrated ions, so the correlations between Al/Fe (*r* = 0.79), Al/Sc (*r* = 0.63), and Fe/Sc (*r* = 0.74) found in our study are the most probable for this reason and were also confirmed in other studies [29]. The synergistic correlation between Zn and Li is known, but in our study, it was of antagonistic character (*r* = −0.41). This could be due to the high content of zinc in plants biofortified with this micronutrient during our experiment. The present study demonstrated that zinc was also positively correlated with other plant micronutrients such as Cu (*r* = 0.56) and Fe (*r* = 0.61). Synergism between zinc and some divalently charged metal ions such as Ni (*r* = 0.63) and Pb (*r* = 0.45) and trivalent Al (*r* = 0.61) suggests that too intensive zinc fertilization can result in increased content of toxic metals. Correlations between other micronutrients were also found (i.e., Fe/Cu *r* = 0.89) in our study. In the literature, positive correlations between Mn, Cu, and Fe can be found [29]. Besides many synergistic, also antagonistic correlations were found, i.e., Cd/Li > Cu/B > Na/Zn > Se/Cd > Zn/Li > Pt/B (*r* < −0.45) while no negative trend was established for typical antagonistic pairs such as Al/Ca and Mn/Ca (*r* = 0.64, *r* = 0.60, respectively) as described in the literature [28]. In our study, the weak correlations between plant mass and the number of germinated seeds (*r* = 0.45) and strong correlations between plant mass and Zn (*r* = 0.76) were found. It was shown that other micronutrients such as Cu (*r* = 0.66) and Fe (*r* = 0.61) and metals Sb (*r* = 0.59) and Cr (*r* = 0.71) are also in synergistic correlation with plant mass. This is the explanation of widespread application of Fe and Cu in plant cultivation which was described in the literature [23, 10].

The differences between results of our study and literature data can be explained by the fertilization leading to biofortification of plants with zinc (elevated Zn(II) doses) which interfere with element content in plants, while in other studies, experiments were carried out on plant standard reference materials, not fertilized [28].

### RGB Analysis

**Fig. 1:**
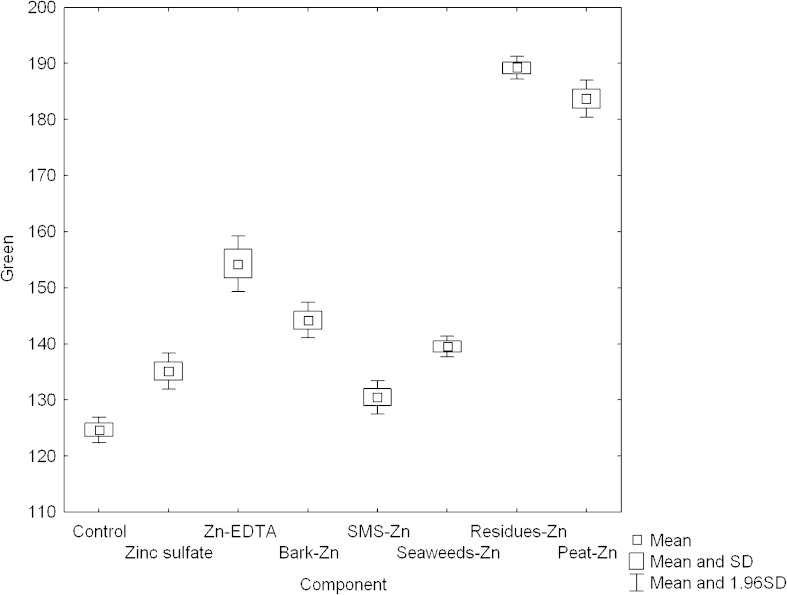
The difference in the intensity of green color in plants fertilized with different materials (normal distribution, *N* = 5)

**Fig. 2:**
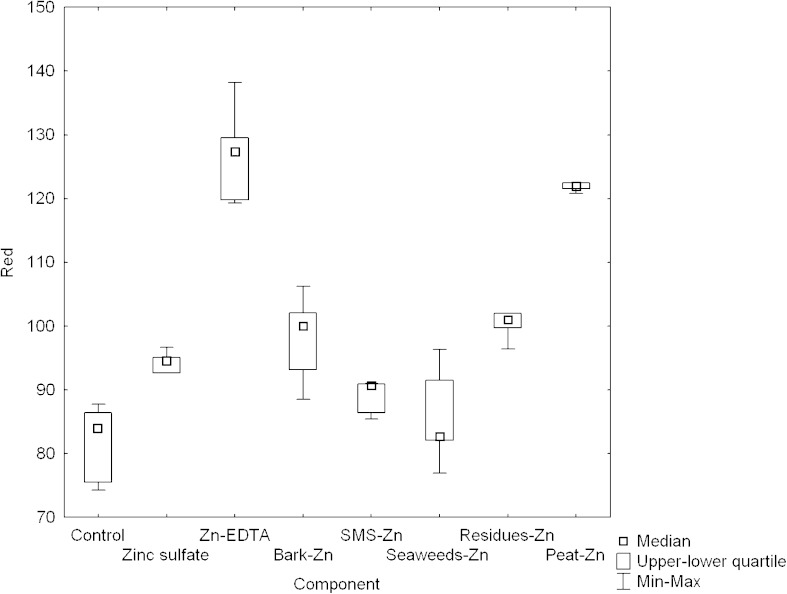
The difference in the intensity of red color in plants fertilized with different materials (other than normal distribution, *N* = 5)

**Fig. 3:**
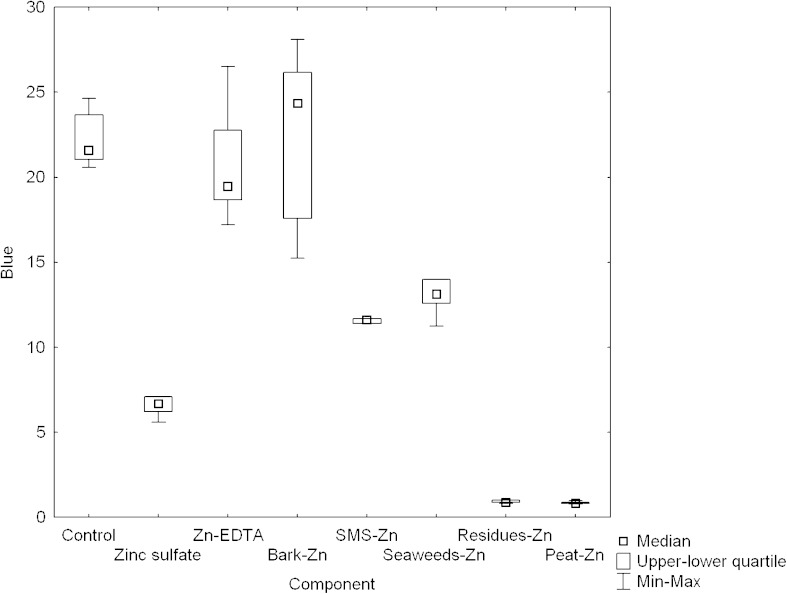
The difference in the intensity of blue color in plants fertilized with different materials (other than normal distribution, *N* = 5)

Leaf color gives a good indication of chlorophyll [28]. There are accepted methods that use image analysis to determine nitrogen and chlorophyll content and crop yield quality [28–32]. In our research, RGB analysis defined as the measurement of the intensity of red, green, and blue colors of microscopic images of leaves was carried out. In the present study, differences between examined groups in intensity of green, red, and blue colors were examined (Figs. 1, 2, and 3). It was shown that the mean value of intensity of green color which, as it is supposed, can be correlated with chlorophyll content in plant, was higher for plants fertilized with new preparations than in the control group in all cases (Fig. 1). Higher value of the intensity of blue color in comparison with control group was observed only for plants fertilized with Zinc sulfate (Fig. 3).

The obtained results of the intensity of red, green, and blue color were used to assess chlorophyll content in examined groups according to methods described in the literature and are presented in Table 5. The obtained results were compared with the proposed method of the estimation of chlorophyll content in plant by the measurement of the intensity of green color. It was shown that similar results could be obtained only with the use of the model basing on logarithmic sigmoid transfer function and the new approach presented here basing on green color analysis [30, 33]. Other methods give divergent results for the same data.

**Table 5:** The comparison of different methods of the correlation between RGB analysis and chlorophyll content in plants for examined plants (*N* = 5)

$\frac{R- B}{R+ B}$ [25]	$\frac{G}{R+ B+ G}$ [30]	$\frac{R}{R+ B+ G}$ [33]	$\frac{G}{R}$ [31]	*R* [32]	*R* + *G* [29]	$\mathrm{logsig}\left(\frac{G-\frac{R}{3}-\frac{B}{3}}{255}\right)$ [28]	*G* [present study]
Peat-Zn 0.986	Residues-Zn 0.650	Zn-EDTA 0.412	Residues-Zn 1.88	Zn-EDTA 127	Peat-Zn 305	Residues-Zn 0.648	Residues-Zn 190
Residues-Zn 0.982	Peat-Zn 0.598	Zinc sulfate 0.403	Seaweeds-Zn 1.69	Peat-Zn 122	Residues-Zn 291	Peat-Zn 0.636	Peat-Zn 183
Zinc sulfate 0.861	Seaweeds-Zn 0.593	Peat-Zn 0.399	Peat-Zn 1.50	Residues-Zn 101	Zn-EDTA 287	Seaweeds-Zn 0.607	Zn-EDTA 160
SMS-Zn 0.772	Zinc sulfate 0.567	SMS-Zn 0.385	Control 1.47	Bark-Zn 98	Bark-Zn 239	Zn-EDTA 0.607	Seaweeds-Zn 144
Seaweeds-Zn 0.729	SMS-Zn 0.565	Bark-Zn 0.374	SMS-Zn 1.47	Zinc sulfate 93	Seaweeds-Zn 230	Bark-Zn 0.599	Bark-Zn 141
Zn-EDTA 0.718	Bark-Zn 0.542	Control 0.365	Bark-Zn 1.45	SMS-Zn 89	Zinc sulfate 225	Zinc sulfate 0.595	Zinc sulfate 132
Bark-Zn 0.634	Control 0.536	Seaweeds-Zn 0.352	Zinc sulfate 1.41	Seaweeds-Zn 86	SMS-Zn 219	SMS-Zn 0.594	SMS-Zn 130
Control 0.570	Zn-EDTA 0.521	Residues-Zn 0.347	Zn-EDTA 1.26	Control 82	Control 202	Control 0.583	Control 120

Shakya et al. [34] showed that the presence of zinc can lead to chlorophyll degradation in plants. In our study, the highest values of the intensity of green color were reported for residues-Zn and peat-Zn. It is suggested that chlorophyll content is not directly dependent on the presence of zinc in fertilizers but it is dependent on the type of component used in plant fertilization. It was reported in the literature that chlorophyll content in plants can be related to the content of other nutrients, i.e., different content of Fe [35].

## Conclusions

The biomass was enriched with Zn(II) ions via biosorption. The utilitarian properties were examined in germination tests. Experiments showed that biological fertilizer components with zinc (seaweeds-Zn, residues-Zn, SMS-Zn) were characterized by higher bioavailability of Zn(II) ions when compared to traditional fertilizers: zinc sulfate and Zn-EDTA. Moreover, it was found that the preparations led to biofortification of plants with this nutrient. The mass of plants fertilized with new preparations was higher than in the control group. It was shown that the mean value of intensity of green color was higher for plants fertilized with new preparations than in the control group.
